# Clinicopathologic Features and Diagnostic Challenges of Mycophenolate Mofetil-Associated Enterocolitis in Lung Transplant Recipients

**DOI:** 10.7759/cureus.116306

**Published:** 2026-09-15

**Authors:** Lucy Shen, Yanping Wang, Andreas Kontosis, Alessa Aragao, Xianzhong Ding, Qiong Zhang

**Affiliations:** 1 Medicine and Surgery, Loyola Stritch School of Medicine, Maywood, USA; 2 Pathology and Laboratory Medicine, Loyola University Medical Center, Maywood, USA

**Keywords:** graft-versus-host disease (gvhd), inflammatory bowel disease, lung transplant, monophosphate dehydrogenase, mycophenolate mofetil colitis

## Abstract

Mycophenolate mofetil (MMF) is a commonly used immunosuppressant for lung transplant recipients. Although it efficiently suppresses transplant rejection and improves graft function, MMF has been found to occasionally cause gastrointestinal (GI) injury. The symptoms are nonspecific and include diarrhea, nausea, vomiting, and rare cases of bleeding. Endoscopic findings range from normal appearance to erythema to ulcers. The histopathologic features mimic several different disorders like graft-versus-host disease (GVHD), cytomegalovirus (CMV) colitis, ischemic colitis, or inflammatory bowel disease (IBD). The nonspecific symptoms, unremarkable endoscopic findings, and overlapping pathological features make the accurate diagnosis of MMF-associated enterocolitis (MAE) very challenging. There are limited case reports regarding MAE in lung transplant patients; however, systematic studies are lacking. Therefore, we analyzed clinical, endoscopic, and pathological features in 12 lung transplant recipients with MAE and aim to improve the recognition of diagnostic challenges.

## Introduction

Mycophenolate mofetil (MMF) is a commonly used immunosuppressive agent in solid organ transplants and autoimmune diseases [1, 2]. Although generally well tolerated, MMF is occasionally associated with a range of gastrointestinal (GI) adverse effects. The exhibited symptoms include watery diarrhea, abdominal pain, nausea, weight loss, and, in rare cases, GI bleeding [3]. In lung transplant patients, the diagnosis may be challenging because these symptoms overlap with other common post-transplant conditions, including infections, graft-versus-host disease (GVHD)-like reactions, and ischemic and inflammatory bowel disease (IBD) [4].

Once taken, MMF is immediately converted to mycophenolic acid (MPA) [5]. MPA suppresses the immune system by blocking the inosine monophosphate dehydrogenase (IMPDH) enzyme, inhibiting the de novo synthesis of guanine nucleotides and leading to the impairment of nucleic acid synthesis. Lymphocytes are mainly dependent on the de novo pathway, which is regulated by IMPDH. Therefore, MMF selectively suppresses immune cell proliferation. MMF-induced GI side effects have been reported in multiple solid organ transplants, such as kidney or liver transplants [6, 7]. However, the exact processes for MMF-induced GI toxicities are still unclear. An acyl glucuronide of MPA (M-2) was found in the plasma of transplant recipients undergoing MMF therapy and may have contributed to the MMF-induced GI side effects due to the pro-inflammatory properties of M-2 [8].

There are several case reports of MMF-induced colitis in lung transplant recipients; however, systematic studies about MMF-associated enterocolitis (MAE) are very limited [9]. In this study, we retrospectively analyzed the clinical, endoscopic, and pathological features of MAE in 12 lung transplant patients. We aim to identify the typical clinical, endoscopic, and pathological features of MAE and explore the association among these findings, thereby shedding light on its diagnostic challenges.

## Materials and methods

Patient selection

This is a retrospective study from a single tertiary medical center (Loyola University Medical Center, Maywood, IL, USA). There were 223 patients with a history of lung transplantation, MMF usage, and GI symptoms, followed by biopsy or resection, identified using electronic medical records between 2008 and 2023. Among these 223 patients, 12 patients who currently received treatment with MMF after lung transplant and exhibited pathologically proven lower GI injury, including enteritis and colitis secondary to MMF, were included in this study. Patients with alternative etiologies for GI injury, including ischemia, GVHD, IBD, or unclear etiologies, etc., were excluded from this study. Patients who showed upper GI injury were also excluded. For infectious workup, patients with current or remote positive serologic cytomegalovirus (CMV) infection were not excluded because the co-infection of CMV was reported in MMF-induced GI injury. The 12 patients showed variable clinical symptoms post transplantation. Endoscopic examination and biopsy were performed in 10 patients. Resections were performed in two patients. The study was reviewed by the Institutional Review Board (IRB) of Loyola University Chicago, Chicago, IL, and was determined to be exempt from IRB review (approval number: 218288).

Chart review

Once selected, the electronic medical records were reviewed for each patient. The demographic features include age, gender, and clinical features, including symptoms, cause of transplant, dose of MMF, duration of MMF use, survival rate, etc., which were analyzed. The endoscopic findings include location, size, and patterns of injury. The histological findings, including location, histological features, and other infections such as CMV, were analyzed and summarized. The histologic findings, including crypt architectural distortion, crypt injury (cryptitis or crypt abscess), crypt dropout, apoptosis, ulcer, and dysplasia, were evaluated and reported. The histologic features were assessed by two independent pathologists with consensus. The findings were descriptive without a scoring system applicable to histologic findings. 

Statistical analysis

Descriptive statistics were used in this retrospective study. Continuous data were expressed as mean ± SD. The categorical variables were expressed as percentages.

## Results

Out of 223 lung transplant recipients who underwent GI tract biopsy or resection, 12 patients (5% incidence) showed pathological findings of MAE. Among these 12 patients, female patients were double compared to male patients (F: M = 2:1). The average age was 61.5 ± 9.5 years. Half of the patients had unilateral and half had bilateral lung transplantation. The most common cause of lung transplantation was chronic obstructive pulmonary disease (58.3%), followed by interstitial lung disease (16.7%) and other causes.

All of the patients had been treated with MMF after the transplantation. The initial dose of MMF ranged from 250 to 1500 mg twice daily, with 1000 mg (58.3%) as the most common initial dose. Some patients had combined immunosuppressive medication, including tacrolimus, methylprednisone, and others. The earliest time to develop MAE is 16 days. Most patients (83%) developed MAE within five years, including 41.7% within the first year after MMF treatment. The longest latency time of MAE was nine years post transplantation. Therefore, the average time to biopsy from MMF exposure was 880 ± 1015 days (range: 16 days to nine years).

The most common presenting GI symptom was diarrhea (75%), followed equally by other symptoms like bleeding, hematochezia, or nausea, etc. Among the 12 patients with MAE, 10 patients underwent endoscopy; nine due to diarrhea and one due to hematochezia. Table 1 summarizes the clinical features.

**Table 1:** Clinical parameters of mycophenolate mofetil-associated enterocolitis

Clinical Parameters	Percentage (%, n=12)
Age	61.5 ± 9.5 years
Gender	
Male	4 (33.3%)
Female	8 (66.7%)
Type of Transplant	
Left Lung	3 (25.0%)
Right Lung	3 (25.0%)
Bilateral Lung	6 (50.0%)
Cause of Lung Transplant	
Chronic Obstructive Pulmonary Disease (COPD)	7 (58.3%)
Interstitial Lung Disease (ILD)	2 (16.7%)
Idiopathic Pulmonary Fibrosis	1 (8.3%)
Polymyositis	1 (8.3%)
Mixed COPD/ILD	1 (8.3%)
Mycophenolate Dose	
250 mg	2 (16.7%)
500 mg	2 (16.7%)
1000 mg	7 (58.3%)
1500 mg	1 (8.3%)
Other Medications	
Tacrolimus	7 (58.3%)
Methylprednisone	6 (50.0%)
Other	4 (33.3%)
None	2 (16.7%)
Time Between Mycophenolate Mofetil Exposure and Gastrointestinal Pathology Diagnosis	
<1 Year	5 (41.7%)
1-5 Years	5 (41.7%)
6-9 Years	2 (16.6%)
Survival Rate	
One-Year Survivability	11 (91.7%)
Five-Year Survivability	5 (41.7%)
Currently Alive	3 (25.0%)
Symptoms	
Diarrhea	9 (75.0%)
Hematochezia	1 (8.3%)
Anemia	1 (8.3%)
Ischemia	1 (8.3%)
Nausea, Vomiting	1 (8.3%)

The survival rate included a one-year survival rate of 91.7% and a five-year survival rate of 41.7%. At the time of the most recent follow-up, nine of the 12 patients were dead. Among the nine deceased patients, one patient died from lung mucinous adenocarcinoma; two patients were lost to follow-up and had an unknown cause of death; and six patients died from the complications of their underlying end-stage diseases.

Endoscopic findings ranged from normal, erythema, and congestion to more severe features such as erosions/ulcers. Table 2 summarizes the endoscopic findings.

**Table 2:** Endoscopic findings of mycophenolate mofetil-associated enterocolitis

Endoscopic Features	Percentage (%, n=10)
Normal	4 (40%)
Erythema	3 (30%)
Ulcers	1 (10%)
Friable Mucosa	1 (10%)
Congestion	1 (10%)

Histological analysis revealed that all cases exhibited chronic mucosal injury characterized by significant crypt injury, including mild architectural distortion and even crypt dropout in about 50% of cases, and marked cryptitis and apoptosis in the majority of patients. In addition, about 17% of patients presented with ulceration. Half of the patients had increased eosinophils in the lamina propria. Notably, lamina propria lymphoplasmacytosis was minimal, distinguishing these cases from inflammatory bowel disease. Meanwhile, immunohistochemistry for CMV was tested in 10 cases and found one positive case of CMV colitis concurrent with MAE. Table 3 and Figure 1 summarize the pathological features.

**Table 3:** Pathologic features of mycophenolate mofetil-associated enterocolitis patients

Pathology	Percentage (%, n=12)
Crypt Injury/Cryptitis	11 (91.7%)
Crypt Dropout	7 (58.3%)
Apoptosis	11 (91.7%)
Ulcer	2 (16.7%)
Eosinophils	6 (50.0%)
Other Infections	1 (8.3%)
Dysplasia	0 (0.0%)
Malignancy	
No	11 (91.7%)
Yes	1 (8.3%)

**Figure 1:**
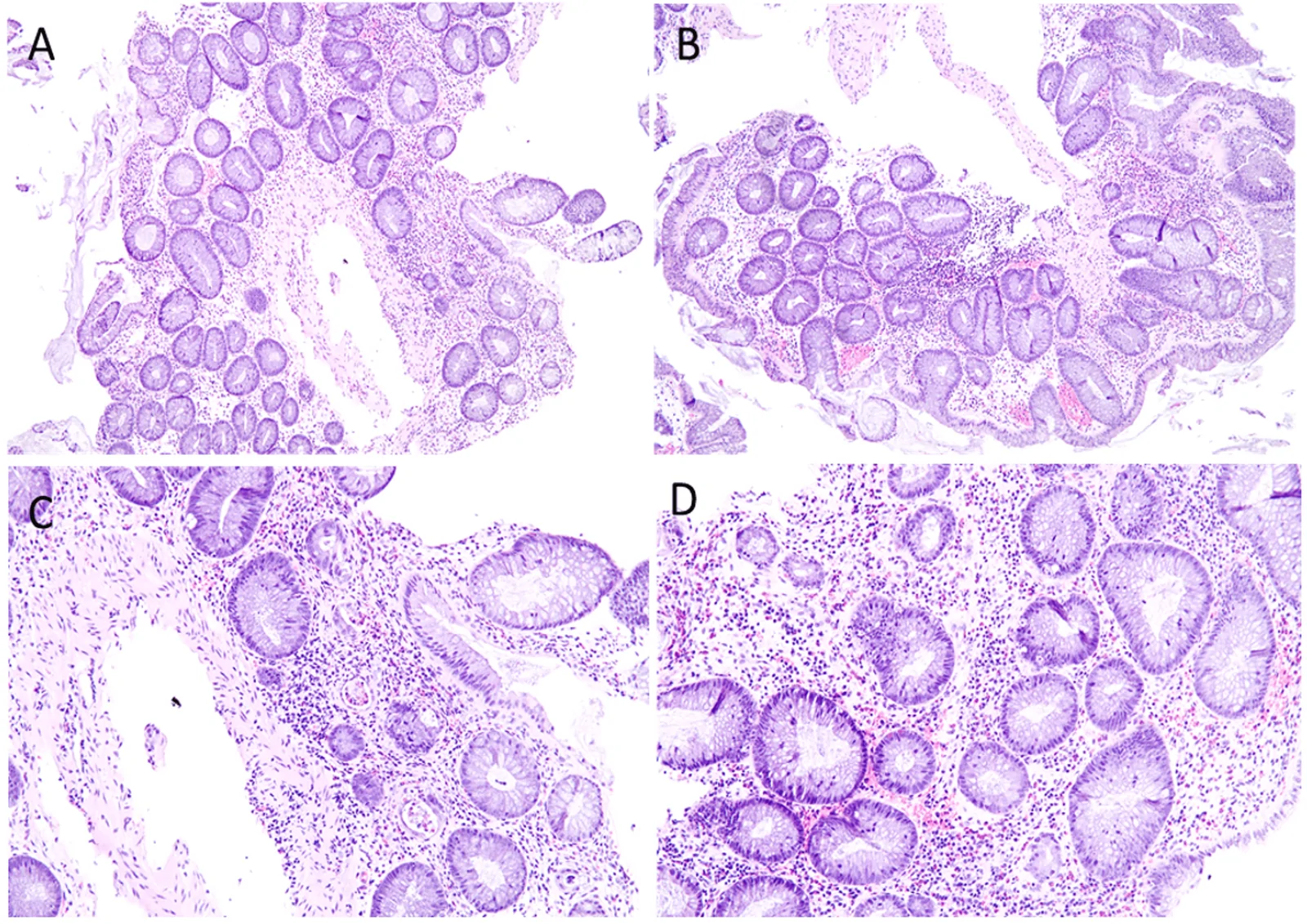
Pathologic features of mycophenolate mofetil-associated enterocolitis A and B: Cryptal architectural distortion and crypt dropout (x200); C: Lamina propria expansion with cryptitis and withered crypts (x400); D: Mixed inflammatory infiltration including lymphocytes, neutrophils, and plasma cells in the lamina propria (x400)

## Discussion

MAE has increasingly been recognized as a GI complication in solid organ transplant recipients, including lung transplant patients. In our study, MAE is more commonly encountered in middle-aged female patients, particularly those who underwent a transplant for chronic obstructive pulmonary disease (COPD). While the exact process remains partially understood, MMF inhibits IMPDH, and this impairs purine synthesis in rapidly dividing cells such as intestinal epithelial cells, which may lead to mucosal damage and epithelial apoptosis. Since lymphocytes are exclusively dependent on this pathway, MPA can exhibit potential immunosuppressive effects through the depletion of T and B cells [10]. Meanwhile, enterocytes are largely dependent on this pathway [11]. This explains why MPA not only exhibits immunosuppression but also induces GI toxicity. A recent study has shown that MMF might change gut microbiota and lead to a secondary bile acid deficiency, whereas supplementation of lithocholic acid alleviated MMF-induced GI toxicity through vitamin D3 receptor (VDR). In addition, a VDR agonist drug, paricalcitol, was found to alleviate MMF-induced GI toxicity [12]. The reason for the high occurrence of MAE in female patients is unclear. Genetic, hormonal, or environmental factors, including vitamin D3 levels, may be responsible for the GI toxicity.

The next finding is that MAE has a broad latency time, ranging from 16 days to nine years. Consequently, it is imperative to clinically consider patients’ MMF usage history since there are overlapping features between MAE and other disorders that cause MAE to be underdiagnosed. Most affected patients received a daily MMF dose of approximately 1,000 mg/day, although toxicity may occur across a range of doses. Importantly, MAE does not necessarily correlate directly with dose intensity, and symptoms may arise despite long-term stable therapy. In many patients, the condition develops relatively early, with approximately 83% presenting within five years of the transplant. However, delayed presentation is well documented, and up to 17% of patients in our study may develop symptoms as late as nine years post transplant. This highlights that MMF toxicity should remain a consideration regardless of the length of time since the transplant.

Similar to the previous reports, the most common symptom was diarrhea, especially watery diarrhea [13]. The main cause of endoscopic examination in 75% of patients was the development of diarrhea. Nausea and vomiting were also found but significantly less than diarrhea. Because diarrhea is common in immunosuppressed transplant patients, symptoms are frequently attributed to infection, antibiotic use, or other medications. It is essential to keep MAE in the differential diagnosis for patients with chronic diarrhea. MMF can induce side effects in both upper and lower GI tracts [14]. This finding reflects that the lower GI tract, especially the right side of the colon, may be the main site of MAE due to the levels of absorption found at the site. With the occurrence of clinical symptoms, a colonoscopy was done on 10 patients to evaluate the status of GI toxicity. Endoscopic findings are variable and may be deceptively subtle. Some patients demonstrated entirely normal mucosa, whereas others showed erythema, erosions, or frank ulcerations. In this study, the most common endoscopic finding was normal appearance (40%), followed by erythema (30%). Few cases exhibited ulcers, friable mucosa, and diverticulosis. Notably, the normal endoscopic finding cannot rule out microscopic pathological abnormalities. A biopsy is necessary for transplant patients with MMF because normal endoscopic findings without a biopsy may potentially delay an MAE diagnosis.

The pathological findings included crypt injuries featuring cryptitis, cryptal atrophy, cryptal architectural distortion, or even crypt dropout, as well as increased neutrophilic or eosinophilic infiltration and increased apoptotic bodies. Histologically, MAE can closely mimic several other pathological processes. Common pathological GI injury patterns with a spectrum of injury patterns mimicking other disorders include the following: 1) Crypt epithelial apoptosis and crypt architectural distortion resembling GVHD [15], 2) Active chronic colitis suggestive of IBD [16], 3) Ulceration with reactive atypia that may raise concern for CMV infection [17] or ischemic colitis [18], and 4) Segmental chronic active inflammation that can overlap with segmental colitis associated with diverticulosis in the left affected colon [19]. Therefore, an accurate diagnosis of MAE requires a comprehensive clinicopathologic correlation, including a thorough clinical history, medication history, and appropriate microbiologic studies. Without this integrated assessment, the diagnosis of MAE can be challenging or even overlooked. The cessation of MMF with alternative immunosuppressive medicines is usually effective. In our study, all patients discontinued MMF and were replaced with alternative immunosuppressive therapy after the diagnosis of MAE. Their GI symptoms were significantly improved afterwards, and no follow-up colonic biopsies were performed. This indicated the importance of MAE diagnosis in the early stage. 

Although this is a retrospective study with a decent number of 12 cases of MMF-induced lower GI injury in lung transplant recipients, there are still several limitations. Firstly, this is a single-center study with a relatively small sample size, which may not fully represent the clinicopathological features. Secondly, the diagnosis of MAE is a diagnosis of exclusion, which lacks clear and definitive histologic criteria for the accurate diagnosis of MAE. Meanwhile, the histologic features are subtle and overlap with other etiologies. Therefore, the histologic interpretation may be influenced by other observers and/or confounded by other causes. Thirdly, the complexities of immunosuppressive agents and immunosuppressive status in lung transplant recipients may include other confounding factors incompletely excluded. Nevertheless, this retrospective study represents one case series of MAE in lung transplant recipients. Furthermore, large-scale and multicentered studies are necessary to clarify the accurate diagnosis of MAE in lung transplant recipients. 

## Conclusions

In conclusion, this study shows that MAE is more likely to affect middle-aged female lung transplant recipients exhibiting watery diarrhea. The disorder may occur not only in the early post-transplant period but also many years after the transplant. Endoscopic findings can be unremarkable, and histological patterns may mimic a variety of infectious and inflammatory conditions. It is essential to maintain MAE in the differential diagnosis to avoid misdiagnosis and facilitate appropriate management, including dose reduction or discontinuation of MMF.
